# SCAMP5 mediates activity-dependent enhancement of NHE6 recruitment to synaptic vesicles during synaptic plasticity

**DOI:** 10.1186/s13041-021-00763-0

**Published:** 2021-03-04

**Authors:** Unghwi Lee, Seung Hyun Ryu, Sunghoe Chang

**Affiliations:** 1 Department of Physiology and Biomedical Sciences, Seoul National University College of Medicine, # 309 Medical Science Bldg, 103 Daehak-ro, Jongno-gu, Seoul, 03080 South Korea; 2 Neuroscience Research Institute, Seoul National University College of Medicine, Seoul, 03080 South Korea

**Keywords:** NHE6, SCAMP5, cLTP, Presynaptic terminal, Activity-dependent synaptic localization, Synaptic vesicle, Presynaptic quantal size, Autism

## Abstract

Na^+^(K^+^)/H^+^ exchanger 6 (NHE6) on synaptic vesicle (SV) is critical for the presynaptic regulation of quantal size at the glutamatergic synapses by converting the chemical gradient (ΔpH) into membrane potential (Δψ) across the SV membrane. We recently found that NHE6 directly interacts with secretory carrier membrane protein 5 (SCAMP5), and SCAMP5-dependent recruitment of NHE6 to SVs controls the strength of synaptic transmission by modulation of quantal size of glutamate release at rest. It is, however, unknown whether NHE6 recruitment by SCAMP5 plays a role during synaptic plasticity. Here, we found that the number of NHE6-positive presynaptic boutons was significantly increased by the chemical long-term potentiation (cLTP). Since cLTP involves new synapse formation, our results indicated that NHE6 was recruited not only to the existing presynaptic boutons but also to the newly formed presynaptic boutons. Knock down of SCAMP5 completely abrogated the enhancement of NHE6 recruitment by cLTP. Interestingly, despite an increase in the number of NHE6-positive boutons by cLTP, the quantal size of glutamate release at the presynaptic terminals remained unaltered. Together with our recent results, our findings indicate that SCAMP5-dependent recruitment of NHE6 plays a critical role in manifesting presynaptic efficacy not only at rest but also during synaptic plasticity. Since both are autism candidate genes, reduced presynaptic efficacy by interfering with their interaction may underlie the molecular mechanism of synaptic dysfunction observed in autism.

## Introduction

NHE6 is an endosomal subtype of monovalent Na^+^(K^+^)/H^+^ exchangers (NHEs) that transports H^+^ ion from the luminal to cytoplasmic part, and Na^+^(K^+^) ion to opposite direction at intracellular vesicles such as endosomes and synaptic vesicles (SVs) [1]. Vacuolar-type H^+^-ATPase and NHE6, with other ion channels/transports on SVs, regulate a proton electrochemical gradient (ΔμH^+^), consisting of the chemical gradient (ΔpH) and membrane potential (Δψ) across the SV membrane to fill SVs with neurotransmitters [2]. Since NHE activity dissipates ΔpH and increases Δψ across the SV membrane at the presynaptic terminals, the uptake of an anionic neurotransmitter such as glutamate into SVs is mainly promoted by Δψ than by ΔpH [3–5]. Since loss-of-function mutations of NHE6 are implicated in various neurodevelopmental, neuropsychiatric and neurodegenerative diseases including autism spectrum disorder (ASD), Christianson syndrome, X-linked intellectual disability, and Alzheimer’s disease, the regulation of NHE6 function and trafficking is critical for underpinning neurophysiological basis of these diseases [1, 6–10].

One of five secretory carrier membrane proteins (SCAMPs) family, SCAMP5 which is enriched in the brain, especially in SVs [11], plays a critical role in SV endocytosis during high neuronal activity [12] and SV release site clearance within the active zone at presynaptic terminals for regulating short-term depression of SV exocytosis [13]. It also has been implicated in several neurodevelopmental and neurodegenerative diseases such as ASD and Parkinson’s disease (PD) [14, 15], SCAMP5 is considered to be engaged in the regulation of function and trafficking of SV proteins for appropriate synaptic transmission.

We recently found that the direct interaction between SCAMP5 and NHE6 regulates the recruitment of NHE6 to SVs for controlling the luminal pH of SVs and subsequent the amount of neurotransmitter release at glutamatergic synapses. In SCAMP5 knockdown (KD) neurons, NHE6 fails to be localized at presynaptic terminals and dispersed along the axon because axonal trafficking of NHE6 is drastically inhibited. The failure of presynaptic localization of NHE6 in SCAMP5 KD neurons leads to the hyperacidification of SVs’ lumen, followed by a significant reduction in the glutamate quantal size at individual presynaptic boutons. Physiological defects caused by SCAMP5 KD are also observed with similar extents in NHE6 knockout neurons, indicating that the decreased glutamate quantal size at the presynaptic side results from disturbed localization of NHE6 to SVs due to the impaired interaction between SCAMP5 and NHE6 in SCAMP5 KD neurons [16].

Activity-dependent neuronal modification carries the changes in synaptic strength including presynaptic release efficacy [17, 18]. Since presynaptic localization of NHE6 is important for presynaptic regulation of glutamate quantal size [16], we hypothesized that modulating NHE6 trafficking to the presynaptic terminals by SCAMP5 could be one of the molecular mechanisms responsible for controlling presynaptic efficacy during synaptic plasticity. Here, we found that the number of NHE6-containing presynaptic boutons was significantly increased by a chemical long-term potentiation (cLTP) in cultured hippocampal neurons and that was completely abrogated by SCAMP5 KD. Interestingly, despite an increase in the number of NHE6-positive boutons, cLTP did not increase the quantal size of glutamate release at the individual presynaptic boutons.

Together with our previous research data [16], these results indicate that the interaction between SCAMP5 and NHE6 is critical for regulating NHE6 localization to SVs during LTP as well as resting state to adjust the presynaptic efficacy at glutamatergic synapses. Since both NHE6 and SCAMP5 are known as the candidate genes for autism, and some of non-sense mutations of NHE6 found in neuropsychiatric disorders fail to associate with SCAMP5 [16], our results suggest an intriguing possibility that defects in NHE6 recruitment into SVs by impaired interaction between NHE6 and SCAMP5 influence the filling of SVs with neurotransmitters, which might underlie the synaptic dysfunction phenotype observed in autism with NHE6 mutations or SCAMP5 reduction.

## Materials and methods

### Plasmid DNA construction

Small hairpin RNA (shRNA) targeting SCAMP5 were made as previously reported [13, 16]. mCherry-tagged synaptophysin plasmid was made from GFP-synaptophysin (kindly provided by Dr. Jane Sullivan, University of Washington). iGluSnFR [19] plasmid was purchased from Addgene (Plasmid #41732). The fidelity of all constructs was verified by DNA sequencing.

### Antibodies

Anti-rabbit SLC9A6 (LS-B13548-50; LifeSpan BioSciences, Seattle, WA) was used in the experiment. Alexa Fluor secondary antibodies were purchased from Thermo Fisher Scientific (Waltham, MA).

### Rat primary hippocampal neuron culture

Hippocampal neurons were prepared as previously described [16, 20]. Briefly, dissociated hippocampal neurons from SD embryonic day 18 fetal rats were treated with papain and plated on poly-d-lysine-coated 18-mm glass coverslips in a 60-mm Petri dish with plating medium (minimum Eagle’s medium; Invitrogen, 0.6% glucose, 1 mM pyruvate, 2 mM l-glutamine, 10% fetal bovine serum; Hyclone) after trituration with a polished half-bore Pasteur pipette. After 3 h, the plating medium was replaced by neurobasal medium (Invitrogen) with 2% B-27 (Invitrogen), 0.5 mM l-glutamine and 4 μM 1-b-d-cytosine-arabinofuranoside (Ara-C; Sigma-Aldrich). The 1/2 of the medium was replaced by a new neurobasal medium with B-27 and l-glutamine at DIV 4, 7 and 14.

### Neuron transfection and image acquisition

Cultured hippocampal neurons were transfected by using the calcium-phosphate method as previously described [13, 16]. Briefly, at DIV 8–10, 6 μg cDNA, 9.3 μl of 2 M CaCl_2_ were mixed in distilled water to a final volume of 75 μl and added to 75 μl 2XBBS (50 mM BES, 280 mM NaCl, and 1.5 mM Na_2_HPO_4_, pH 7.1). The mixture solution was incubated for 20 min at room temperature and added to neurons in transfection medium (minimum Eagle’s medium, 1 mM pyruvate, 0.6% glucose, 10 mM l-glutamine, and 10 mM HEPES, pH 7.65) for 50–60 min at 37 ℃, 5% CO_2_ incubator. After incubation, the transfection medium was replaced with washing medium (minimum Eagle’s medium, 1 mM pyruvate, 0.6% glucose, 10 mM l-glutamine, and 10 mM HEPES, pH 7.35) for 20–30 min at 37 ℃, 5% CO_2_ incubator and again changed with the original neurobasal medium. Transfected neurons on coverslips were mounted and imaged by Olympus IX71 fluorescence microscope, a 60× 1.35 N.A. oil-immersion lens using an Andor Zyla-5.5-CL3 sCMOS camera (Andor Technologies, Belfast, Northern Ireland) driven by MetaMorph imaging software (Universal Imaging Corporation, West Chester, PA). Images were analyzed by ImageJ/FIJI (NIH, Bethesda, MD) software.

### Immunocytochemistry

For immunostaining NHE6 proteins, neurons were fixed in 3% glyoxal with 20% absolute ethanol and 7.5% acetic acid for 1 h at room temperature and quenched with 100 mM NH_4_Cl solution for 30 min at room temperature. After that, cells were permeabilized and blocked with 2.5% BSA and 0.1% Triton X-100/PBS for 15 min at room temperature. Cells were incubated with primary antibodies in blocking solution for 3 h at 37 ℃ and with Alexa Fluor-conjugated secondary antibody in blocking solution for 45 min at 37 ℃. After immunostaining, cells on coverslips were mounted on a slide glass and fluorescent images were acquired.

### Image acquisition of glutamate release using iGluSnFR

iGluSnFR signals were obtained as previously described [16, 21]. Briefly, transfected neurons with iGluSnFR at DIV 14–16 were mounted in a perfusion/stimulation chamber (Chamlide, South Korea) on the stage of a Nikon Eclipse Ti-U fluorescence microscope (Nikon, Tokyo, Japan) with a 40× and 1.0 N.A. oil-immersion lens at 35 ℃ in Tyrode’s solution (136 mM NaCl, 2 mM CaCl_2_, 2.5 mM KCl, 2 mM MgCl_2_, 10 mM glucose, 10 mM HEPES, pH 7.4, 285–290 mOsm). Stream images were obtained at 100 Hz with an Andor iXon 897 EMCCD camera (Andor Technologies) with cropped mode, driven by MetaMorph Imaging Software (Universal Imaging Corporation). To measure the iGluSnFR response of spontaneous glutamate release, 12 trials of 500 frames (100 Hz imaging acquisition) were acquired with a 60 s interval in the presence of 1 μM TTX to prevent neuronal firing.

### Induction of cLTP and FM 1–43 uptake/destaining

cLTP was induced by forskolin in cultured hippocampal neurons as previously described [22]. Briefly, forskolin (50 μM) or DMSO (as a control) was treated to cultured hippocampal neurons at DIV 14–16 during 30 min in at 37 ℃ CO_2_ incubator for inducing cLTP. After that, FM 1–43 loading/unloading experiment was performed or endogenous NHE6 protein was immunostained. For FM 1–43 uptake, neurons at DIV14–16 were stimulated with 300 APs at 10 Hz in the presence of 10 μM FM 1–43 (Invitrogen, Carlsbad, CA) and 50 μM APV in Tyrode’s solution (136 mM NaCl, 2 mM CaCl_2_, 2.5 mM KCl, 2 mM MgCl_2_, 10 mM glucose, 10 mM HEPES, pH 7.4, 285–290 mOsm), kept in additional 30 s after stimulation for labeling post-stimulus endocytosed synaptic vesicles. Cells were washed with low-Ca^2+^ and high-Mg^2+^ Tyrode’s solution (119 mM NaCl, 0.5 mM CaCl_2_, 2.5 mM KCl, 10 mM MgCl_2_, 30 mM glucose, 25 mM HEPES, pH 7.4, 285–290 mOsm) with 1 mM ADVASEP-7 (Sigma-Aldrich, ST. Louis, MO) for the removal of cell surface-bound FM 1–43 dye. After washing period (5 min), 1200 APs at 10 Hz were applied to neurons for destaining of loaded FM 1–43. The imaging was obtained by Olympus IX71 fluorescence microscope, a 60× 1.35 N.A. oil-immersion lens using an Andor Zyla-5.5-CL3 sCMOS camera (Andor Technologies) driven by MetaMorph imaging software (Universal Imaging Corporation) in closed perfusion/stimulation chamber (Chamlide) and perfused continuously with respective solution at 35 ℃ by using MPS-8 multi-valve perfusion system (Chamlide) at 0.5–1.0 mL/min. Images were analyzed by ImageJ/FIJI (NIH) software and decay time constant (τ) was analyzed and calculated by fitting with single exponential function by using Origin 9 software (OriginLab).

### Statistical analysis

For analysis of protein colocalization, Manders’ colocalization coefficients (MCC) were calculated using ImageJ/FIJI (NIH) with the JACoP plug-in function (https://imagej.nih.gov/ij/plugins/track/jacop.html). Average of MCC value of each sample was compared by One-way ANOVA with LSD post hoc test. Values are indicated as mean ± SEM. Quantitative measurements of FM 1–43 and iGluSnFR responses from individual boutons were obtained using ImageJ/FIJI (NIH). Individual rectangular regions of interest (ROIs) were drawn around the synaptic boutons and marked by hand, then obtained by averaging pixel intensities of selected areas and average intensities were calculated. Large puncta, the clusters of smaller synapses, were excluded from the selection procedure. The center of intensity of each synapse was calculated to correct for any image shifts throughout the experiment. Fluorescence was expressed in intensity units that correspond to fluorescence values averaged over all pixels within the region of interest. Data were collected from 20 to 30 boutons in each coverslip and “n” stands for the number of boutons or coverslips in FM 1–43 and iGluSnFR experiments. iGluSnFR data were presented as mean ± SD compared by paired samples *t*-test and analyzed by using Origin 9 software (OriginLab Corporation, Northampton, MA) and SPSS Statistics 23 (IBM, Armonk, NY). Significance of all data is reported as *p < 0.05, **p < 0.01, ***p < 0.001.

## Results

### Chemical LTP induced by forskolin increases presynaptic release efficacy and the number of functional presynaptic boutons

Our recent data showed that SCAMP5, which is highly enriched in SVs and involved in vesicular membrane trafficking, regulates the recruitment of NHE6 to SVs by their direct interaction [16]. Since presynaptic efficacy is enhanced by activity-dependent neuronal modification [23] and the proper localization of NHE6 is important for presynaptic regulation of glutamate quantal size [16], we wondered that an increase in synaptic activity would affect NHE6 recruitment to the presynaptic terminals. To address this possibility, we induced a cLTP in cultured hippocampal neurons by using forskolin, an adenylyl cyclase activator, which is known to induce presynaptic LTP by activating cAMP/PKA signaling pathway [18, 24–26].

**Fig. 1:**
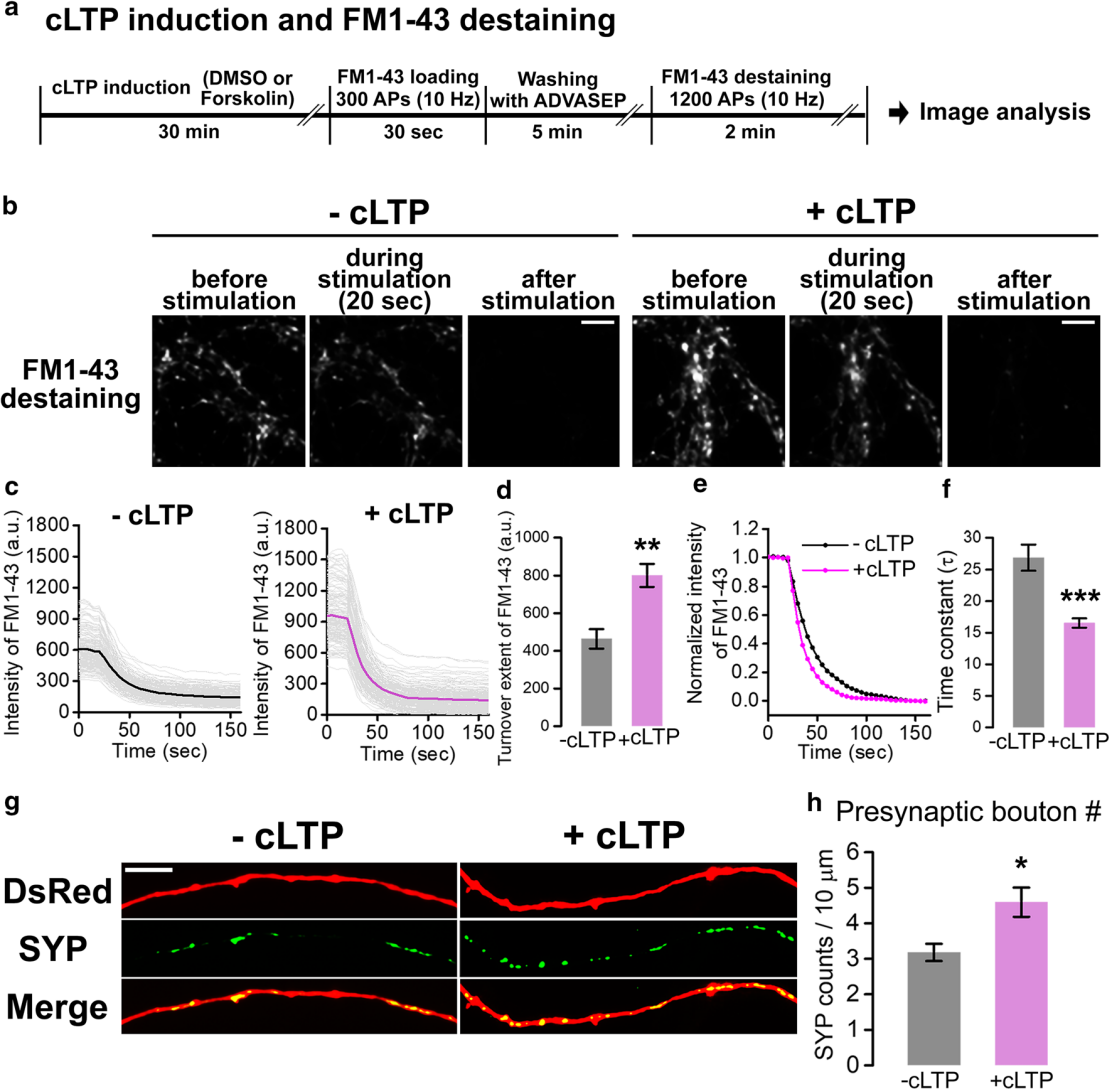
Forskolin-induced cLTP enhances presynaptic activity in cultured hippocampal neurons. **a** Experimental procedure of imaging and analyzing FM 1–43 uptake and destaining after cLTP induction by forskolin or DMSO application. **b** Representative images of cultured hippocampal neurons at DIV14 during FM 1–43 destaining in control (− cLTP) and cLTP-induced (+ cLTP) neurons. Scale bar: 10 μm. **c** Average fluorescent intensity of FM 1–43 during destaining period with or without cLTP induction. Traces of individual response (light gray lines) and average trace (thick colored lines) of the FM 1–43 signal at presynaptic boutons after treatment of DMSO (− cLTP, n = 170 boutons in 6 coverslips) or forskolin (+ cLTP, n = 167 boutons in 6 coverslips). **d** Turnover extent of FM 1–43 during destaining period. − cLTP, 463.629 ± 51.637 a.u., n = 6 (coverslips); + cLTP, 800.752 ± 61.040 a.u., n = 6 (coverslips), p = 0.002, analyzed by Student’s *t*-test. **e** Normalized average fluorescent intensity of FM 1–43 during destaining period with or without cLTP induction. **f** Time constant (τ) of FM 1–43 intensity during destaining period. − cLTP, 26.871 ± 2.054 s, n = 6 (coverslips); + cLTP, 16.543 ± 0.737 s, n = 6 (coverslips), p = 0.0008, analyzed by Student’s *t*-test. **g** Representative images of cultured hippocampal neurons co-transfected with DsRed and GFP-synaptophysin (SYP) with or without cLTP induction. Scale bar: 5 μm. **h** The number of presynaptic boutons per 10 μm axon. − cLTP, 3.179 ± 0.242 counts/10 μm, n = 6 (coverslips); + cLTP, 4.594 ± 0.411 counts/10 μm, n = 6 (coverslips), p = 0.021, analyzed by Student’s *t*-test. Values are indicated as mean ± SEM. a.u., arbitrary unit. *p < 0.05, **p < 0.01, ***p < 0.001

To evaluate the induction of a cLTP in cultured neurons, we performed *N*-(3-triethylammoniumpropyl)-4-(4-(dibutylamino)styryl) pyridinium dibromide (FM1–43) loading and destaining assay after treatment of forskolin or dimethyl sulfoxide (DMSO). Cultured hippocampal neurons at days in vitro (DIV) 14–16 were stimulated with 300 action potentials (APs) at 10 Hz in the presence of FM 1–43 and were washed for the removal of cell surface-bound FM 1–43. After washing period, neurons were stimulated with 1200 APs at 10 Hz for destaining of loaded FM 1–43 (Fig. 1a).

We found that the application of forskolin to cultured hippocampal neurons increased the amount of uptake of FM 1–43, and accelerated its destaining rate, which represents the increase in recycling SV pool size and the release probability of SV release (Fig. 1b–f). In addition, forskolin treatment significantly increased the number of presynaptic boutons (Fig. 1g, h). In our previous study, using FM 1–43 and retrospective immunostaining of a postsynaptic SH3 and multiple ankyrin repeat domains (SHANK), we confirmed that the newly formed presynaptic boutons were functional, and were juxtaposed to SHANK-positive postsynaptic structures, indicating that cLTP increases the number of bona fide functional synapses [22].

### cLTP significantly increases the number of NHE6-positive presynaptic boutons, which is completely inhibited by SCAMP5 KD

**Fig. 2:**
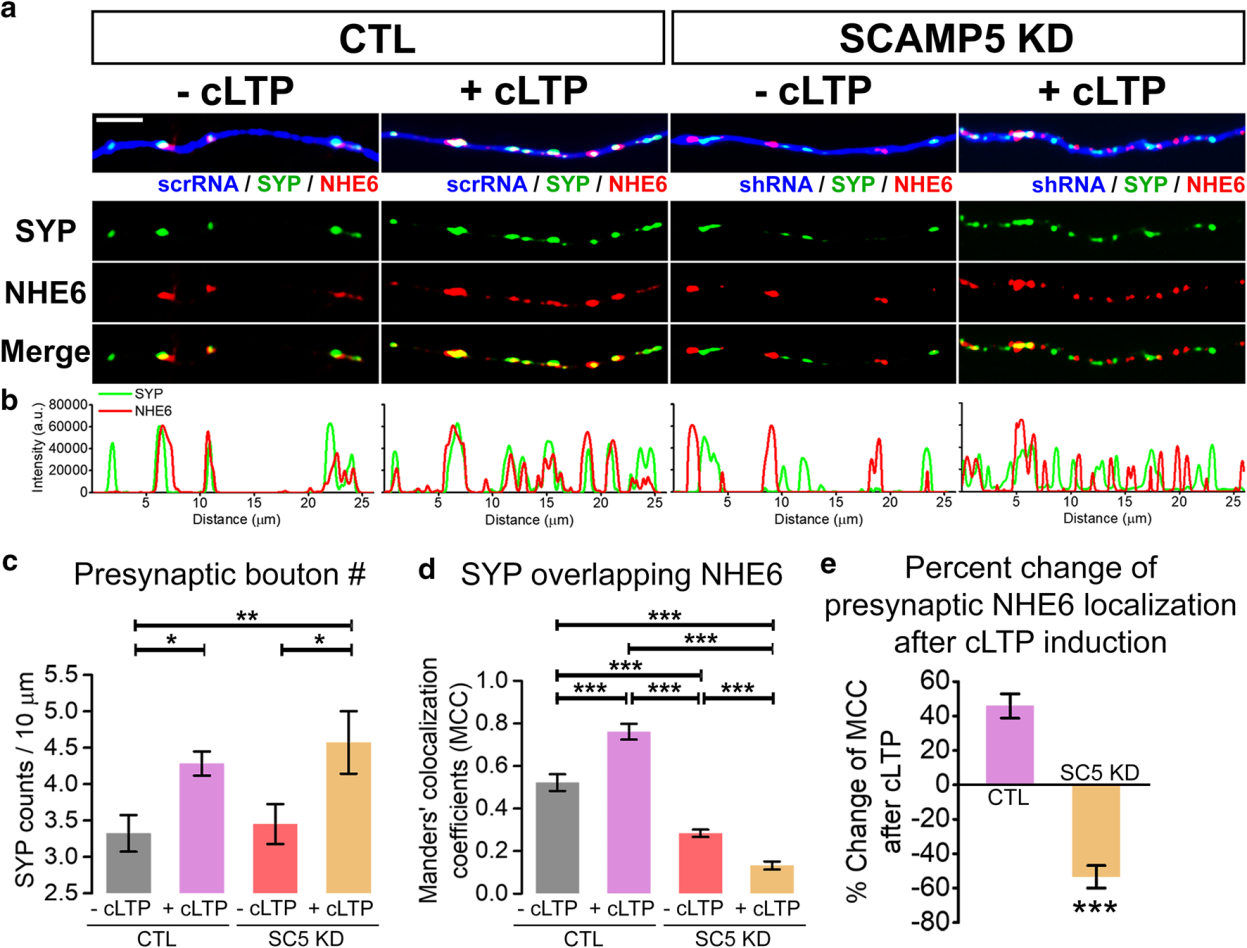
Activity-dependent presynaptic recruitment of NHE6 is completely inhibited by SCAMP5 KD. **a** Representative images of cultured hippocampal neurons co-transfected with BFP-scrRNA or -shRNA SCAMP5, GFP-synaptophysin (SYP) and endogenous NHE6 proteins were immunostained (NHE6) after treatment of forskolin (+ cLTP) or DMSO (− cLTP). Scale bar: 5 μm. **b** Fluorescent intensity profiles of the respective protein along the axon. a.u., arbitrary unit. **c** The number of presynaptic boutons per 10 μm axon. CTL with − cLTP, 3.323 ± 0.251 counts/10 μm, n = 10 (coverslips); CTL with + cLTP, 4.282 ± 0.166 counts/10 μm, n = 7 (coverslips); SC5 KD with − cLTP, 3.451 ± 0.274 counts/10 μm, n = 10 (coverslips); SC5 KD with + cLTP, 4.572 ± 0.429 counts/10 μm, n = 7 (coverslips), F_(3, 30)_ = 4.354, p = 0.012, analyzed by one-way ANOVA test followed by LSD post hoc test. **d** Manders’ colocalization coefficients of SYP overlapping NHE6. CTL with − cLTP, 0.522 ± 0.039, n = 5 (coverslips); CTL with + cLTP, 0.761 ± 0.037, n = 5 (coverslips); SC5 KD with − cLTP, 0.284 ± 0.017, n = 7 (coverslips); SC5 KD with + cLTP, 0.132 ± 0.019, n = 6 (coverslips), F_(3, 19)_ = 98.777, p = 9.028E−12, analyzed by one-way ANOVA test followed by LSD post hoc test. **e** Percent change of presynaptic NHE6 localization extent after cLTP induction. CTL, + 45.841 ± 7.048%, n = 5 (coverslips); SC5 KD, − 53.451 ± 6.551%, n = 6 (coverslips), p = 2.8E−06, analyzed by Student’s *t*-test. Values are indicated as mean ± SEM. *p < 0.05, **p < 0.01, ***p < 0.001

To investigate whether the recruitment of NHE6 to the presynaptic terminals is altered in an activity-dependent manner and this mechanism is mediated by SCAMP5, cultured hippocampal neurons were co-transfected with synaptophysin (SYP) and small hairpin RNA (shRNA)-targeting SCAMP5 to suppress the endogenous SCAMP5 expression or scrambled RNA (scrRNA) as a control (Fig. 2a) and endogenous NHE6 proteins were immunostained after cLTP induction.

The number of presynaptic boutons was increased in both control and SCAMP5 KD neurons (Fig. 2a–c), indicating that SCAMP5 KD did not affect cLTP-induced increase in the number of presynaptic boutons. When counting the number of SYP overlapping with NHE6, there is a significant increase in SYP/NHE6 overlap in control neurons (0.522 ± 0.039 in − cLTP of CTL; 0.761 ± 0.037 in + cLTP of CTL, Fig. 2a–e). This means that NHE6 was recruited not only to the existing presynaptic boutons that previously had no NHE6, but also to the newly formed presynaptic boutons during cLTP induction. In contrast, the colocalization extent between SYP and NHE6 was significantly decreased in SCAMP5 KD neurons after cLTP induction (0.132 ± 0.019 in + cLTP of SC5 KD, Fig. 2a–e). Since most of NHE6 already failed to colocalize with SYP in SCAMP5 KD neurons before cLTP induction (0.284 ± 0.017 in − cLTP of SC5 KD, Fig. 2a, b, d) [16], our results suggest that activity-dependent increase in NHE6 recruitment to the pre-existing or newly formed presynaptic terminals is completely inhibited in SCAMP5 KD neurons.

### cLTP does not increase the quantal size of glutamate release at individual presynaptic boutons

**Fig. 3:**
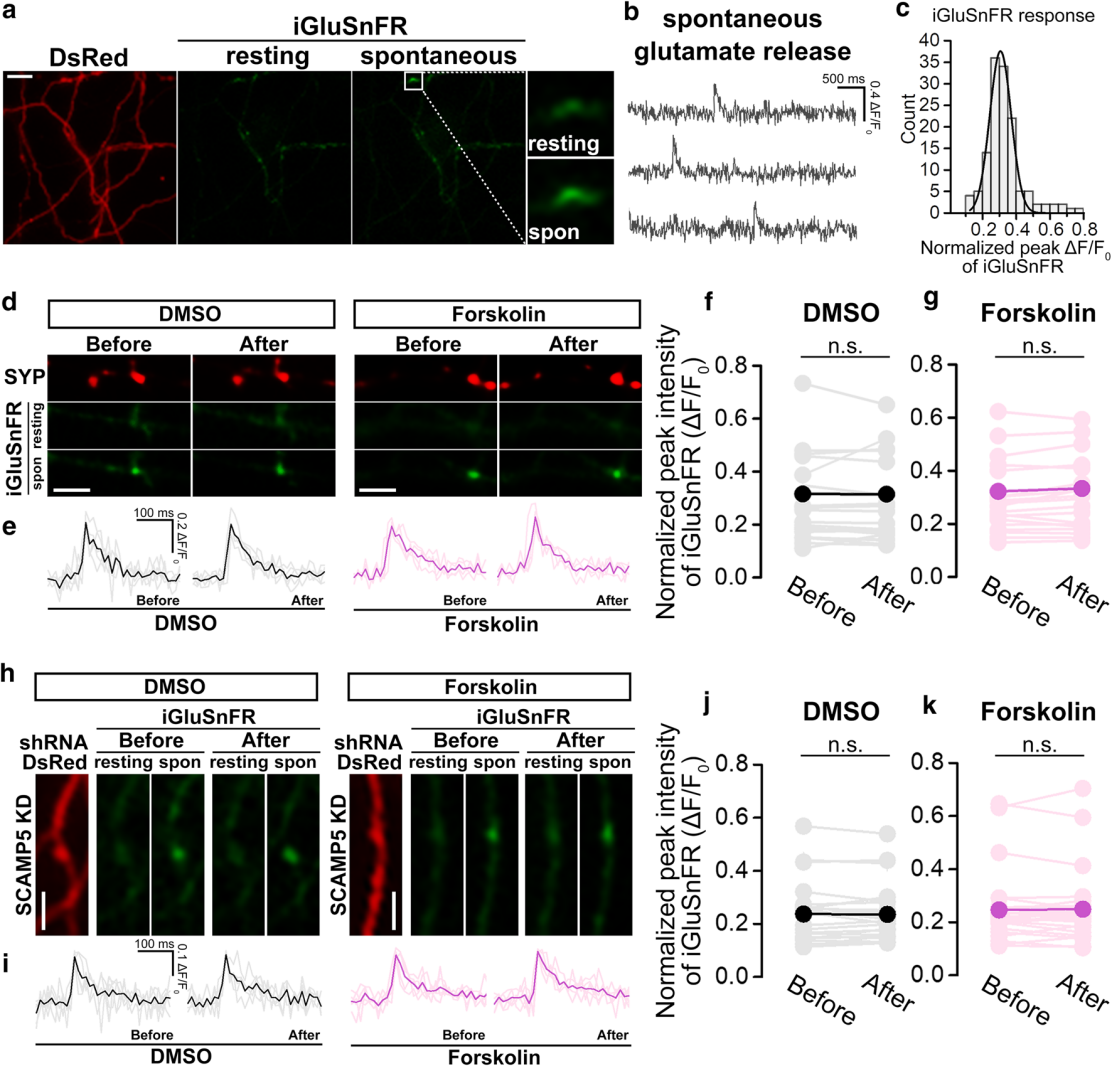
Forskolin-induced cLTP does not change glutamatergic quantal release at individual presynaptic boutons in cultured hippocampal neurons. **a** Representative images of cultured hippocampal neurons co-transfected with DsRed and iGluSnFR. Images of iGluSnFR represent resting condition and in the response of glutamate released spontaneously. Scale bar: 5 μm. **b** Representative traces of the iGluSnFR signal during spontaneous glutamate release at one presynaptic bouton. **c** Amplitude histogram graph of spontaneous iGluSnFR response, n = 135 (boutons). The graph was fitted with a single Gaussian distribution. **d, h** Representative images of cultured hippocampal neurons co-transfected with mCherry-synaptophysin (SYP) or DsRed-shRNA SCAMP5, and iGluSnFR. Response signal of iGluSnFR due to spontaneous glutamate release was obtained before and after treatment of DMSO or Forskolin, respectively. Scale bar: 5 μm. **e, i** Representative traces of individual response (thin colored lines) and average trace (thick colored lines) of the iGluSnFR signal during spontaneous glutamate release at one presynaptic bouton before and after treatment of DMSO or Forskolin, respectively. **f, g** Changes of normalized peak intensity of iGluSnFR due to spontaneous glutamate release before and after treatment of DMSO or Forskolin in control neurons. Before (DMSO), 0.316 ± 0.226 ΔF/F_0_, n = 20 (boutons, 3 coverslips); After (DMSO), 0.315 ± 0.225 ΔF/F_0_, n = 20 (boutons, 3 coverslips). p = 0.954, Before (Forskolin), 0.322 ± 0.177 ΔF/F_0_, n = 20 (boutons, 3 coverslips); After (Forskolin), 0.333 ± 0.182 ΔF/F_0_, n = 20 (boutons, 3 coverslips). p = 0.075, analyzed paired samples *t*-test. **j, k** Changes of normalized peak intensity of iGluSnFR due to spontaneous glutamate release before and after treatment of DMSO or Forskolin in SCAMP5 KD neurons. Before (DMSO), 0.237 ± 0.113 ΔF/F_0_, n = 24 (boutons, 3 coverslips); After (DMSO), 0.236 ± 0.104 ΔF/F_0_, n = 24 (boutons, 3 coverslips). p = 0.772, Before (Forskolin), 0.246 ± 0.129 ΔF/F_0_, n = 28 (boutons, 3 coverslips); After (Forskolin), 0.249 ± 0.130 ΔF/F_0_, n = 28 (boutons, 3 coverslips). p = 0.670, analyzed paired samples *t*-test. Values are indicated as mean ± SD. *n.s.* non-significance. *p < 0.05, **p < 0.01, ***p < 0.001

We also wondered whether increasing NHE6 recruitment to SVs by cLTP increases the glutamate quantal size at individual synapses because the NHE6 recruitment to SVs determines the amount of glutamate uptake into SVs, and cation/H^+^ exchange activity is known to augment Δψ to a greater extent than predicted by the V-ATPase alone [3]. Thus, the presynaptic quantal size would be expected to increase as NHE6 recruitment to an individual SVs increases. To address this possibility, we used iGluSnFR, a fluorescent glutamate sensor, that reflects the amount of glutamate released at individual presynaptic boutons [19]. Using iGluSnFR, we have recently measured the amount of glutamate released spontaneously or in response to a few action potentials at the individual presynaptic boutons and such that we were able to avoid the postsynaptic interference for quantal size variations. Thus, the only source of quantal size variation we could detect with iGluSnFR is presynaptic, and the extent of iGluSnFR response is a reliable indicator of the quantal size of glutamate release [16, 21]. The amplitude histogram of spontaneous iGluSnFR responses was fitted with a single Gaussian distribution (Fig. 3a–c), indicating that the unit response was reliably detected. We then co-transfected iGluSnFR along with shRNA SCAMP5 or SYP into cultured hippocampal neurons and analyzed the amplitude of iGluSnFR signal by spontaneously released glutamate before and after cLTP induction (Fig. 3d, h). We found that the peak intensity of the iGluSnFR response to spontaneous glutamate release did not change before and after cLTP in control neurons (Fig. 3d–g). In SCAMP5 KD, although the amount of glutamate released spontaneously was significantly lower than in the control as consistent with our recent results [16], the peak intensity of iGluSnFR response also did not change after cLTP (Fig. 3h–k), indicating that SCAMP5 KD inhibited NHE6 localization not only at rest but also during cLTP. All these results suggest that despite an increase in the number of NHE6-positive boutons by cLTP, the amount of glutamate released at individual presynaptic boutons remains unaltered.

## Discussion

In this study, we showed that SCAMP5-mediated NHE6 recruitment to the presynaptic terminals was significantly increased during cLTP. Since we recently showed that the direct interaction between SCAMP5 and NHE6 plays a critical role in the recruitment of NHE6 to SVs, which regulates the luminal pH of SVs and subsequent the amount of neurotransmitter release at glutamatergic synapses at rest [16], our current results suggest that SCAMP5-mediated NHE6 recruitment is critical for presynaptic regulation of glutamate quantal size not only at rest but also during LTP. These results further raised the possibility that the enhanced presynaptic recruitment of NHE6 by SCAMP5 could be one of the molecular determinants of presynaptically-expressed synaptic plasticity.

We, however, found that despite the increase in NHE6 recruitment to the presynaptic boutons during cLTP, the glutamate quantal size at the individual presynaptic boutons did not change after cLTP (Fig. 3). Several feasible mechanisms can be speculated to explain these results. First, the steady-state levels of ΔμH^+^ across the SV membrane can be maintained mainly by the V-ATPase. If V-ATPase sets the upper limit of ΔμH^+^ generated in the given SV, even if more NHE6 is recruited to individual SVs after cLTP induction, no further increase in the Δψ, thus the quantal size remained unaffected. Second, the presence of NHE6 in SVs might set the lower limit of glutamate uptake, and a certain number of NHE6 in the individual SVs could be sufficient to fully uptake glutamate into SVs. It is noteworthy, however, that the overexpression of vesicular glutamate transporter 1 is known to lead to excess glutamate uptake [27, 28]. In addition, an increase in presynaptic cation concentration above a resting value either artificially or by enhancing hyperpolarization-activated cyclic nucleotide-gated (HCN) channel activation increases the quantal size [4]. Both indicate that V-ATPase activity may not be a rate-limiting step for glutamate uptake into SVs. Furthermore, it is known that cation/H^+^ exchange activity augments Δψ to a greater extent than predicted by the V-ATPase alone [3]. Thus, the lack of changes in the amount of spontaneous glutamate release after cLTP induction may indicate that the additional NHE6 is not recruited to the presynaptic boutons that already had NHE6. We, however, do not rule out the possibility that NHE activity affects the rate constant for Δψ, thus more NHE6 only facilitates reaching the steady-state Δψ but does not affect the steady-state levels of Δψ. Although intriguing, since nothing has been known about the kinetics of NHE activity related to V-ATPase activity on Δψ in living cells, this certainly requires further study.

We are aware that cLTP in culture conditions cannot fully reflect what happens during LTP in vivo, but many previous results using cLTP in culture have provided plenty of significant insights on our understanding of synaptic physiology [17, 22, 24] and have later been proven to occur similarly in vivo as well [29–35]. Thus, further study is required to find out whether SCAMP5/NHE6 interaction plays the role during LTP formation or maintenance in vivo. Also, LTP primarily has been considered to involve the postsynaptic phenomena, however, various research groups suggested that LTP also induced the presynaptic changes including the increased recycling pool of SVs, the enhanced release probability, and the increased number of presynaptic boutons [17, 18, 22, 24, 25, 29–35]. We also found that forskolin-induced cLTP caused presynaptic changes listed above (Fig. 1), indicating that forskolin-induced cLTP in cultures reflects most, if not all, presynaptic changes caused by LTP in brain slices.

Our current results together with our recent findings [16] suggest that SCAMP5 may function directly or indirectly with axonal trafficking mechanisms such as regulating biophysical properties or the motor proteins, post-translational modification of the microtubule, and the activation of motor adaptor proteins for controlling recruiting-process of the SV proteins to presynaptic terminals. However, the underlying molecular mechanisms are currently unknown. Thus, further study for investigating this detailed process is necessary and this will shed light on the question about the mechanisms of presynaptic localization of SV proteins that are regulated by SCAMP5 or other SCAMPs interaction.

SCAMPs are expressed ubiquitously and reside on secretory vesicles for regulating the exocytosis and endocytosis of intracellular recycling vesicles [11], and play a role in vesicular trafficking from the trans-Golgi network to regulate the recycling process of endosomal compartments [36–42]. One of SV-enriched SCAMPs family, SCAMP5, was mainly studied as one of regulators for presynaptic function [12, 13], however, it was also previously reported that SCAMP5 was localized at Golgi-associated compartments [43, 44] and acts as a facilitator of cytokine secretion, and was interacted with synaptotagmin 1 [44]. This indicates that SCAMP5 may have other binding partners at Golgi apparatus in neurons and those interactions can regulate vesicular and protein trafficking from Golgi compartments to its cellular destinations for neuronal development or synaptic transmission.

Recent genetic analyses have reported two SCAMP5 mutations, R91W and G180W. R91W was identified from patients with pediatric epilepsy and juvenile PD [14], and G180W was identified from patients with ASD, intellectual disability and seizures [45]. The residue of SCAMP5 R91 and G180 is located in the 2/3 loop and C-terminal cytoplasmic domain, respectively, and those SCAMP5 mutants caused the alteration in protein expression level, subcellular distribution, and binding properties compared to those of wild-type SCAMP5, and resulted in the neuronal dysfunction with excessive excitatory neurotransmission [14]. Especially, since SCAMP5 R91 residue is located in the 2/3 loop domain in which NHE6 [16], adaptor protein complex 2 (AP-2), and clathrin [13] can bind to, the pathological phenotype caused by SCAMP5 R91W mutant may have resulted from the alteration in the extent of protein interaction with its binding partners, followed by the physiological dysfunction from defects in neurotransmission. It is certainly of interest and requires further study.

## Data Availability

All experimental protocols are described in “Materials and methods” section or in the references therein, and resources are available upon request from the corresponding author SC.

## Abbreviations

NHE: Na^+^(K^+^)/H^+^ exchanger
SV: Synaptic vesicle
SCAMP: Secretory carrier membrane protein
cLTP: Chemical long-term potentiation
ASD: Autism spectrum disorder
PD: Parkinson’s disease
cAMP: Cyclic adenosine monophosphate
PKA: Protein kinase A
FM 1–43: *N*-(3-Triethylammoniumpropyl)-4-(4-(dibutylamino)styryl) pyridinium dibromide
DMSO: Dimethyl sulfoxide
DIV: Days in vitro
AP: Action potential
shRNA: Small hairpin ribonucleic acid
KD: Knockdown
SYP: Synaptophysin
iGluSnFR: Intensity-based glutamate-sensing fluorescent reporter
V-ATPase: Vacuolar-type H^+^-ATPase
AP-2: Adaptor protein complex 2
